# Non-Monotonic Compressive Strength of Cement Mortars with Alternative Fine Aggregates

**DOI:** 10.3390/ma19163437

**Published:** 2026-08-13

**Authors:** Feng Ji, Yuexiang Xing, Jing Fu, Hui Yin, Gang Wang

**Affiliations:** 1 School of Mathematics and Physics, Xinjiang Institute of Engineering, Urumqi 830023, China; jf11@xjie.edu.cn (F.J.); 15022912661@163.com (Y.X.); 2 Key Laboratory of Xinjiang Coal Resources Green Mining, Ministry of Education, Xinjiang Institute of Engineering, Urumqi 830023, China; 3 Xinjiang Coal Design and Research Institute Co., Ltd., Urumqi 830091, China; 18199157953@163.com; 4 Graduate School, Xinjiang Institute of Engineering, Urumqi 830023, China; 18999277520@163.com; 5 School of Energy Engineering, Xinjiang Institute of Engineering, Urumqi 830023, China

**Keywords:** alternative fine aggregate, cement mortar, preliminary screening, non-monotonic strength development, strength retention, SVI_7–28_

## Abstract

Alternative fine aggregates are often assessed using compressive strength at a single reference age, which may conceal an early maximum followed by later strength loss. This preliminary screening study compared mortars containing river sand (RS), standard sand (StS), desert sand (DS), soil sand (SS), coal gangue sand (CGS), and metamorphic rock sand (MRS) under one nominal mixture design. For each aggregate, one mortar batch was prepared and nine 70.7 mm cubes were cast, with three specimens tested at 3, 7, and 28 d. RS, StS, DS, and SS continued to gain strength. Within the single CGS batch, strength decreased from 15.56 ± 0.31 MPa at 7 d to 11.68 ± 0.15 MPa at 28 d; within the single MRS batch, it decreased from 21.63 ± 0.48 MPa to 15.75 ± 0.20 MPa. The corresponding losses were 24.95% and 27.18%, and exploratory within-batch Tukey tests yielded *p* < 0.001. These statistics describe specimen-level variation within the tested batches and do not establish batch-to-batch reproducibility. Representative 28 d SEM fields, raw-aggregate EDS, and qualitative XRD provide contextual observations but lack the temporal and spatial resolution needed to reconstruct a defect-formation process between 7 and 28 d. Because aggregate moisture state, absorption, flow, air content, and compaction were not independently controlled, the results identify a screening signal that requires independent-batch validation rather than a general material mechanism.

## 1. Introduction

Natural river sand is a major constituent of mortar and concrete, but intensive extraction can disturb river morphology, aquatic habitats, and local sediment balances. Sustainable management of sand resources has therefore become an international concern [1,2,3]. Candidate substitutes include desert sand, recycled fine aggregate, crushed-rock fines, industrial by-products, and locally available soil-derived sands [4,5,6,7,8,9]. Their performance cannot be inferred from origin alone because grading, particle shape, surface texture, clay-bearing fines, mineral composition, moisture condition, and water absorption influence the behavior of both fresh and hardened materials.

Aggregate moisture is particularly important when materials are compared at a fixed nominal water-to-cement ratio. Porous particles can absorb some of the mixing water, whereas moisture carried into the batch increases the total water content. Both processes alter the free water available for cement hydration and flow. Poon et al. [10] showed that aggregate moisture condition affects slump and compressive strength, and later studies demonstrated that mixing sequence and surface treatment can modify the behavior of highly absorptive aggregates [11,12]. Very fine or clay-bearing particles can further increase water demand and reduce the effectiveness of polycarboxylate-based admixtures [4,5,13].

The aggregate–paste interfacial transition zone (ITZ) also governs load transfer. Depending on particle roughness, mineralogy, packing, and local water availability, the ITZ may contain compositional gradients, relatively large pores, oriented crystals, or weakly bonded regions [14,15,16,17]. Continued cement hydration usually increases solid volume and refines capillary pores, but paste densification does not necessarily increase bulk strength when interfacial gaps, microcracks, or weak aggregate particles interrupt the load-bearing network [18,19,20,21].

Most studies of alternative fine aggregates report strength at standard ages and emphasize the 28 d endpoint [22,23,24,25]. Far less attention has been given to materials that reach an early maximum and then lose strength within the same 28 d curing period. This behavior matters in material screening because an acceptable early-age result may not indicate stable later-age performance. Its interpretation is difficult when moisture condition, workability, compaction, aggregate porosity, and interfacial development are not independently controlled.

This preliminary screening study examines the strength trajectories of six mortars prepared with identical nominal proportions. The response from 7 to 28 d is summarized by SVI_7–28_ so that early load-bearing capacity and subsequent retention can be considered together. Mechanical performance is compared with aggregate properties, selected 28 d SEM observations, raw-aggregate EDS, and qualitative XRD. The objective is to identify aggregate systems that warrant controlled follow-up testing, not to establish batch-to-batch reproducibility or a general mechanism from the present single-batch dataset.

## 2. Materials and Methods

### 2.1. Raw Materials

Portland composite cement (P.C 42.5R) conforming to GB 175–2023 [26] was supplied by Xinjiang Qingsong Jianhua Chemical Industry (Group) Co., Ltd., Alar City, China. Its chemical composition and physical properties are listed in Table 1.

**Figure 1:**
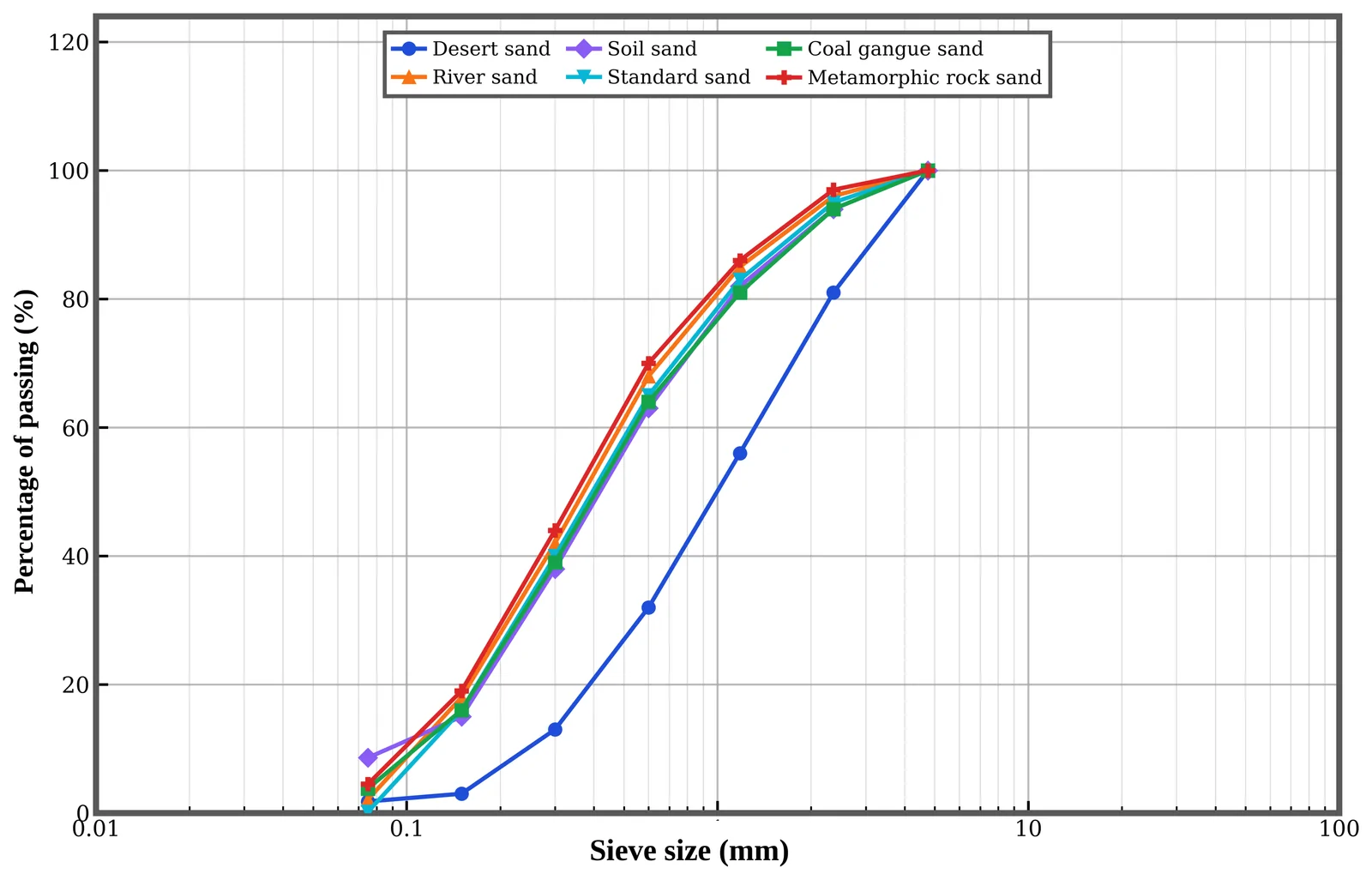
Particle-size distribution curves of the cement and fine-aggregate raw materials.

**Figure 2:**
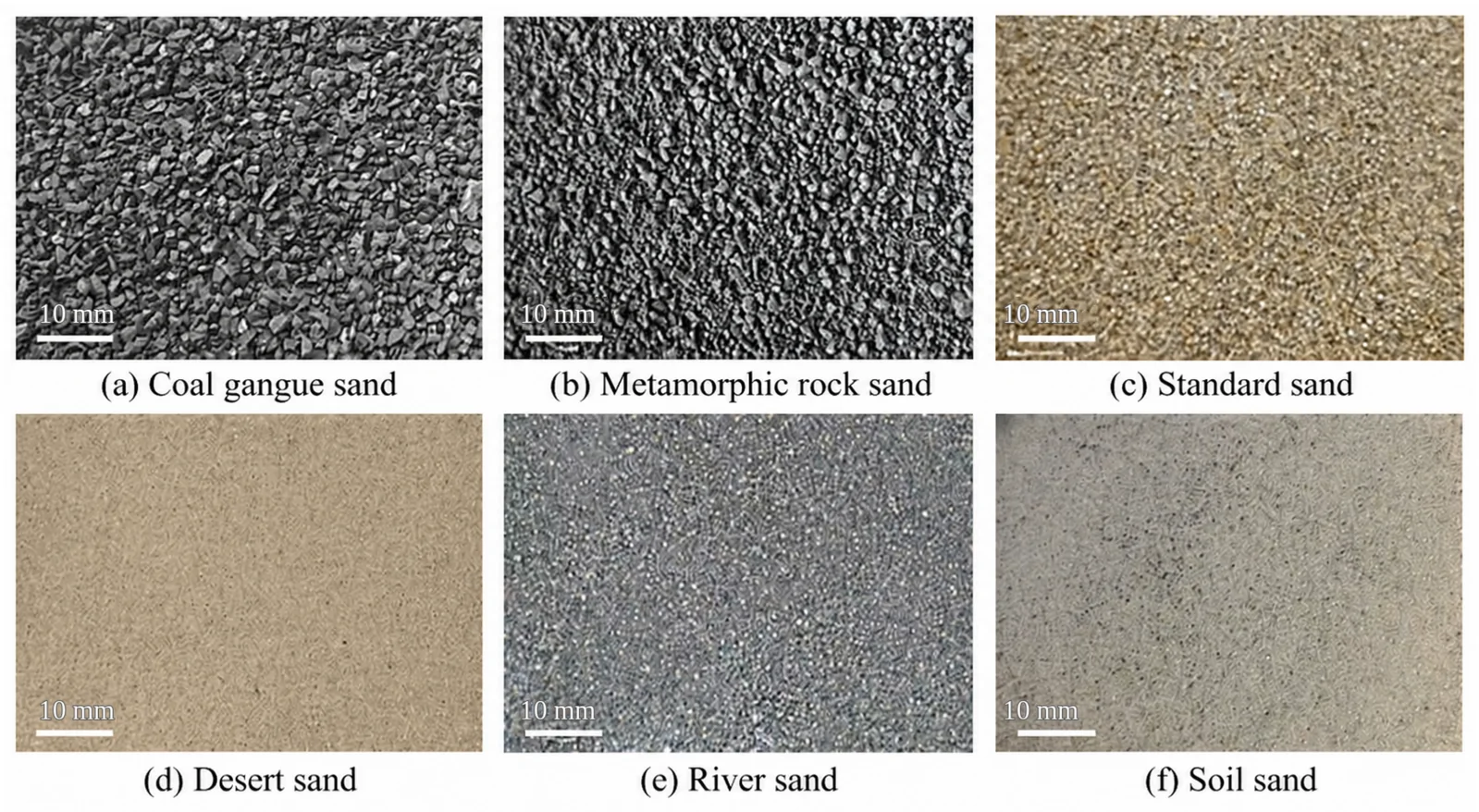
Representative appearances of the six fine aggregates: (**a**) CGS, (**b**) MRS, (**c**) StS, (**d**) DS, (**e**) RS, and (**f**) SS.

Six fine aggregates were investigated: river sand (RS), standard sand (StS), desert sand (DS), soil sand (SS), coal gangue sand (CGS), and metamorphic rock sand (MRS). Except for the StS, the materials were collected from Urumqi and surrounding areas in Xinjiang, China. The StS was a certified reference sand produced in Xiamen, China. Particle-size distributions and representative appearances are shown in Figure 1 and Figure 2.

The MRS was a manufactured fine crushed-rock aggregate produced from gneiss obtained as a by-product of a local crushed-stone plant in the Urumqi region. The parent rock underwent jaw crushing, impact crushing, and screening to below 4.75 mm. The screened fraction was washed to remove loosely adhered dust and then air-dried. For the CGS, the reported laboratory protocol included use at its measured moisture content without an additional conditioning or surface-treatment step before mortar mixing. MRS was included because it was locally available at the study site; it is not presented as a universally abundant or inherently more sustainable substitute for river sand.

RS and StS were dominated by SiO_2_, whereas SS and CGS contained larger proportions of Al- and Fe-bearing components. DS had the highest bulk CaO content among the natural sand materials. CGS showed the highest measured porosity and the lowest bulk density, while MRS had relatively low porosity and the largest fineness modulus. Tap water was used for mixing and curing. The polycarboxylate-based superplasticizer had a solid content of 20%; the manufacturer and city/country of origin were not available in the archived experimental records.

### 2.2. Mixture Proportions and Specimen Preparation

The cement:fine aggregate:mixing water:commercial superplasticizer solution ratio was 1:2.87:0.45:0.015. The aggregate:cement ratio was close to the conventional 1:3 mortar proportion, and the same formulation was retained for all six materials to provide a common screening comparison. The admixture dosage corresponded to approximately 0.30% active solids by cement mass. Individual mixtures were not optimized for equal flow or maximum strength. For each aggregate type, a single mortar batch was prepared; nine cubes were cast from that batch, and three cubes were allocated to each of the 3, 7, and 28 d tests. No independently repeated mixing batch was prepared for any aggregate.

Cement and fine aggregate were blended without water for 2 min. Water and superplasticizer were then added, and mixing continued until the mortar appeared homogeneous. The mortar was placed in 70.7×70.7×70.7 mm cube molds and compacted on a vibrating table. Specimens were covered after casting, demolded after 24 h, and cured at (20±2) °C and relative humidity above 95% until testing.

The aggregates were used at their measured laboratory moisture content, and neither saturated-surface-dry conditioning nor aggregate-specific water correction was applied. Flow, air content, and short-term absorption were not measured. The nominal water-to-cement ratio was therefore identical, but the effective water available during mixing and curing may have differed among aggregates. This limitation is considered explicitly in the interpretation.

### 2.3. Compressive Strength and Statistical Analysis

Compressive strength was measured at 3, 7, and 28 d using 70.7 mm mortar cubes with reference to JGJ/T 70–2009 [27]. A YAW-2000D computer-controlled electrohydraulic servo compression testing machine (Jinan Hensgrand Instrument Co., Ltd., Jinan, China) with a nominal capacity of 2000 kN and Class 1 load accuracy (±1.0%) was used. A load was applied continuously at 1.5 kN/s. Each cube was centered between the platens, and the maximum failure load was recorded.

Three specimens were tested for each aggregate–age condition. Cube compressive strength was calculated as

(1) $$f_{m,cu}=K\frac{N_{u}}{A},$$

where $f_{m,cu}$ is the compressive strength (MPa), $N_{u}$ is the maximum failure load (N), A=70.7×70.7=4998.49 mm^2^ is the loaded area, and K=1.35 is the conversion coefficient specified in Clause 9.0.4 of JGJ/T 70–2009 [27]. The same coefficient was applied formulaically to every individual specimen, irrespective of aggregate type, curing age, or observed result, before group summaries and statistical analyses were produced. For example, the StS 28 d specimen with $N_{u}=245439$ kN gave $f_{m,cu}=1.35\times 245439/4998.49=66.29$ MPa. No replicate was removed. Individual failure loads and calculated strengths for all 54 specimens are provided in Supplementary Table S1; results in the main text are reported as the mean ± sample standard deviation.

Curing-age effects within each aggregate system were evaluated by one-way analysis of variance followed by the Tukey honestly significant difference test. The criterion for statistical significance was set at p<0.05. Because the three age groups for each aggregate were specimen-level subsamples from one mortar batch, these analyses were treated as exploratory within-batch comparisons. They quantify differences relative to the scatter among three cubes at each age but do not test batch-to-batch reproducibility or provide a complete estimate of population variability. The magnitude of the change and the within-group coefficient of variation were therefore also considered.

The strength variation index from 7 to 28 d was defined as

(2) $${\mathrm{SVI}}_{7–28}=\frac{f_{28}-f_{7}}{f_{7}}\times 100\%,$$

where $f_{7}$ and $f_{28}$ are the mean strengths at 7 and 28 d. Positive values indicate strength gain, and negative values indicate strength loss. SVI_7–28_ is a descriptive index for the present comparison, not an intrinsic material constant.

### 2.4. SEM, EDS, and XRD Characterization

Fragments were taken from the interiors of 28 d mortar specimens, dried at 60 °C, and sputter-coated with gold. SEM was conducted with a JEOL JSM-IT500 microscope (Tokyo, Japan). Fields from DS, CGS, and MRS mortars are presented in the main text because DS retained positive strength development, whereas CGS and MRS showed negative SVI_7–28_. The fracture-and-drying procedure can create or enlarge cracks and pores; the images were therefore used primarily for qualitative comparison.

The quantitative panel accompanying the SEM images contains two-dimensional field descriptors. Dark pore regions were segmented under a common thresholding procedure, and crack traces were calibrated from the image scale bars and reduced to line skeletons. The pore area fraction was calculated as the segmented pore area divided by the analyzed image area. Crack density was calculated as total traced crack length divided by image area. The values describe the analyzed fields only. They are not equivalent to bulk porosity or three-dimensional crack density, and no inferential statistics were applied because replicate-field data were unavailable.

Raw MRS and CGS particles were also examined by SEM-EDS at an accelerating voltage of 15 kV. Representative electron images, elemental overlays, individual maps, sum spectra, and area-scan compositions were obtained (EDS software version unavailable; related website: https://www.jeol.com; accessed 12 August 2026). Carbon was retained in the maps and spectra but excluded from the bar-chart comparison because the signal may be influenced by carbon tape or coating. EDS results are local, semi-quantitative elemental mass fractions, whereas Table 2 reports normalized bulk oxide mass fractions. The two datasets therefore use different compositional bases and cannot be compared numerically without an element-to-oxide conversion, which was not performed. EDS was not used to identify hydration phases or quantify ITZ bond strength.

XRD was performed with a Rigaku SmartLab diffractometer (Tokyo, Japan) using Cu Kα radiation (λ=1.5406 Å) at 40 kV and 50 mA. The specimens comprised raw MRS, raw CGS, MRS mortar at 3 and 28 d, and CGS mortar at 28 d. Dried material was crushed and ground to below 80 μm. Patterns were collected from 5 to 80° in 2θ with a step size of 0.01° and a scanning rate of 10°/min. Traces were vertically offset where necessary to improve legibility; peak positions were unchanged. Phase assignments were qualitative, and no internal standard, amorphous-phase quantification, or Rietveld refinement was applied [28,29].

## 3. Results

### 3.1. Compressive-Strength Evolution

**Figure 3:**
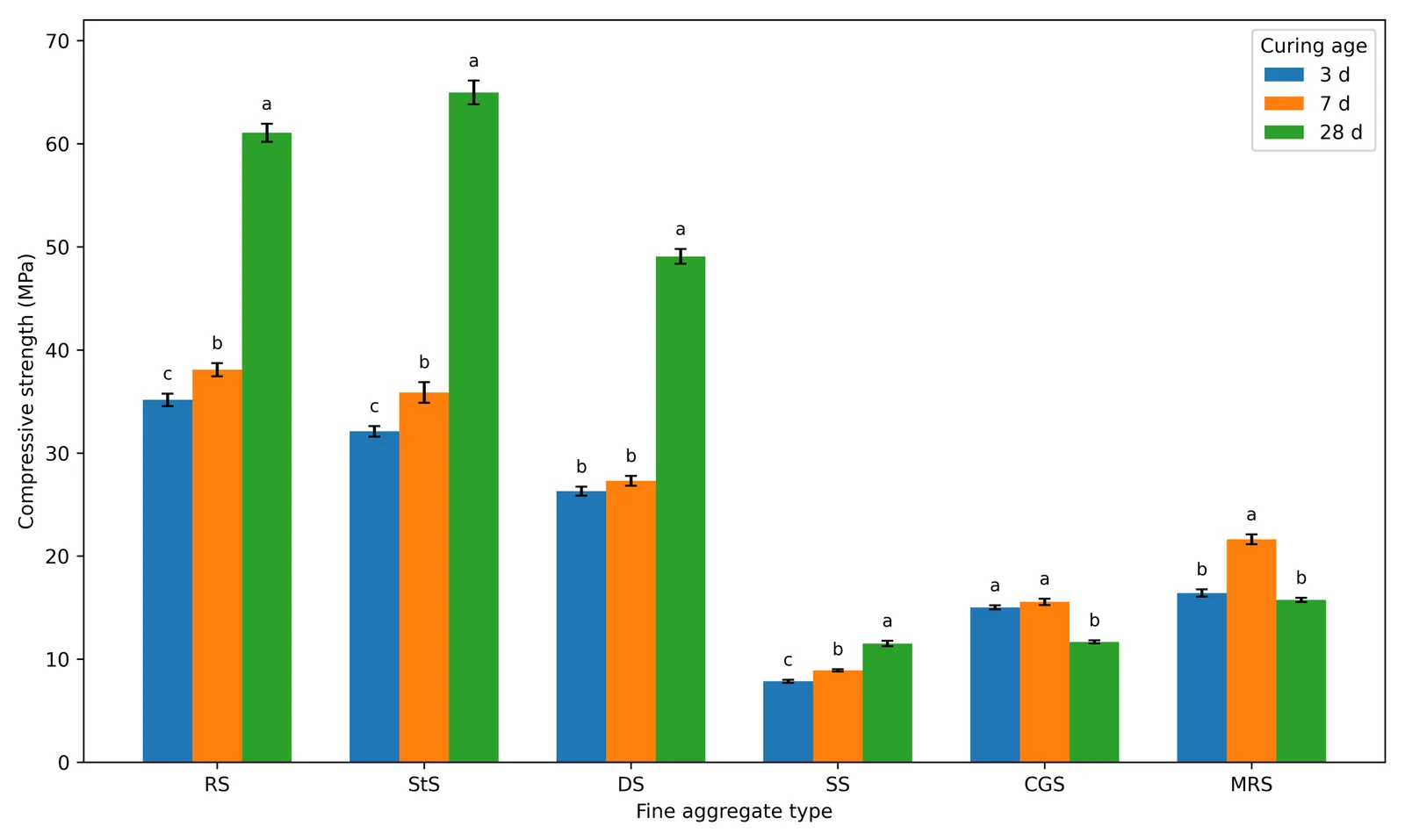
Compressive strength of mortars prepared with different fine aggregates at 3, 7, and 28 d. Columns and error bars represent the mean and sample standard deviation of three specimen-level subsamples from one batch per aggregate. Different lowercase letters within one aggregate type indicate differences among ages in the exploratory within-batch Tukey HSD comparison (p<0.05); they do not establish batch-to-batch reproducibility.

Figure 3 presents the mean strength and sample standard deviation of the three cubes in each aggregate–age subgroup. RS, StS, DS, and SS increased between 3 and 28 d. Within the single MRS batch, strength increased from 16.42±0.36 MPa at 3 d to 21.63±0.48 MPa at 7 d (exploratory within-batch Tukey test, p<0.001), then decreased to 15.75±0.20 MPa at 28 d (p<0.001 relative to 7 d). The 3 and 28 d MRS values were not significantly different within this comparison (p=0.145).

Within the single CGS batch, strength changed from 15.03±0.20 MPa at 3 d to 15.56±0.31 MPa at 7 d; this within-batch difference was not significant (p=0.066). It then decreased to 11.68±0.15 MPa at 28 d (p<0.001 relative to both 3 and 7 d). The coefficients of variation for CGS were 1.97% at 7 d and 1.28% at 28 d; the corresponding values for MRS were 2.23% and 1.29%. These values describe consistency among the three tested cubes at each age only. They do not demonstrate reproducibility across independently prepared batches.

### 3.2. Strength Retention Beyond 7 d

**Figure 4:**
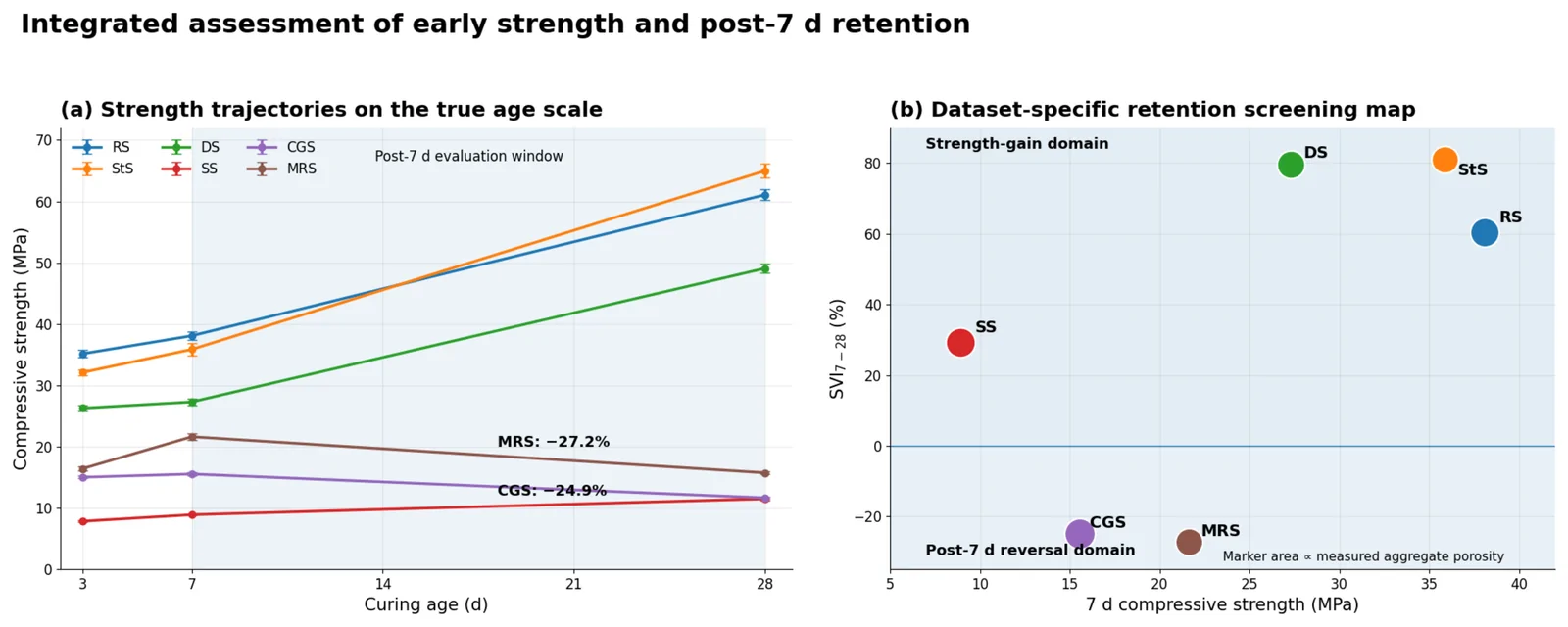
Strength development and post-7 d retention. (**a**) Compressive-strength trajectories plotted against the true curing-age scale; error bars denote the sample standard deviation of three specimen-level subsamples from one batch per aggregate. (**b**) Dataset-specific map combining 7 d strength and SVI_7–28_; marker area is proportional to measured aggregate porosity. The map is descriptive, does not establish independent-batch reproducibility, and does not imply that porosity alone controls strength evolution.

The SVI_7–28_ values were 60.33%, 81.12%, 79.71%, 29.18%, −24.95%, and −27.18% for RS, StS, DS, SS, CGS, and MRS, respectively. Figure 4b separates the mechanical level reached at 7 d from the direction of the subsequent response. CGS and MRS entered the negative-retention region despite their markedly different measured porosities of 55.9% and 38.4%. Bulk porosity alone, therefore, cannot explain the reversal. The figure does not interpolate the unmeasured behavior between 7 and 28 d and does not define a universal acceptance boundary.

## 4. Microstructure and Phase Characterization

### 4.1. SEM Morphology and Image-Based Defect Descriptors

**Figure 5:**
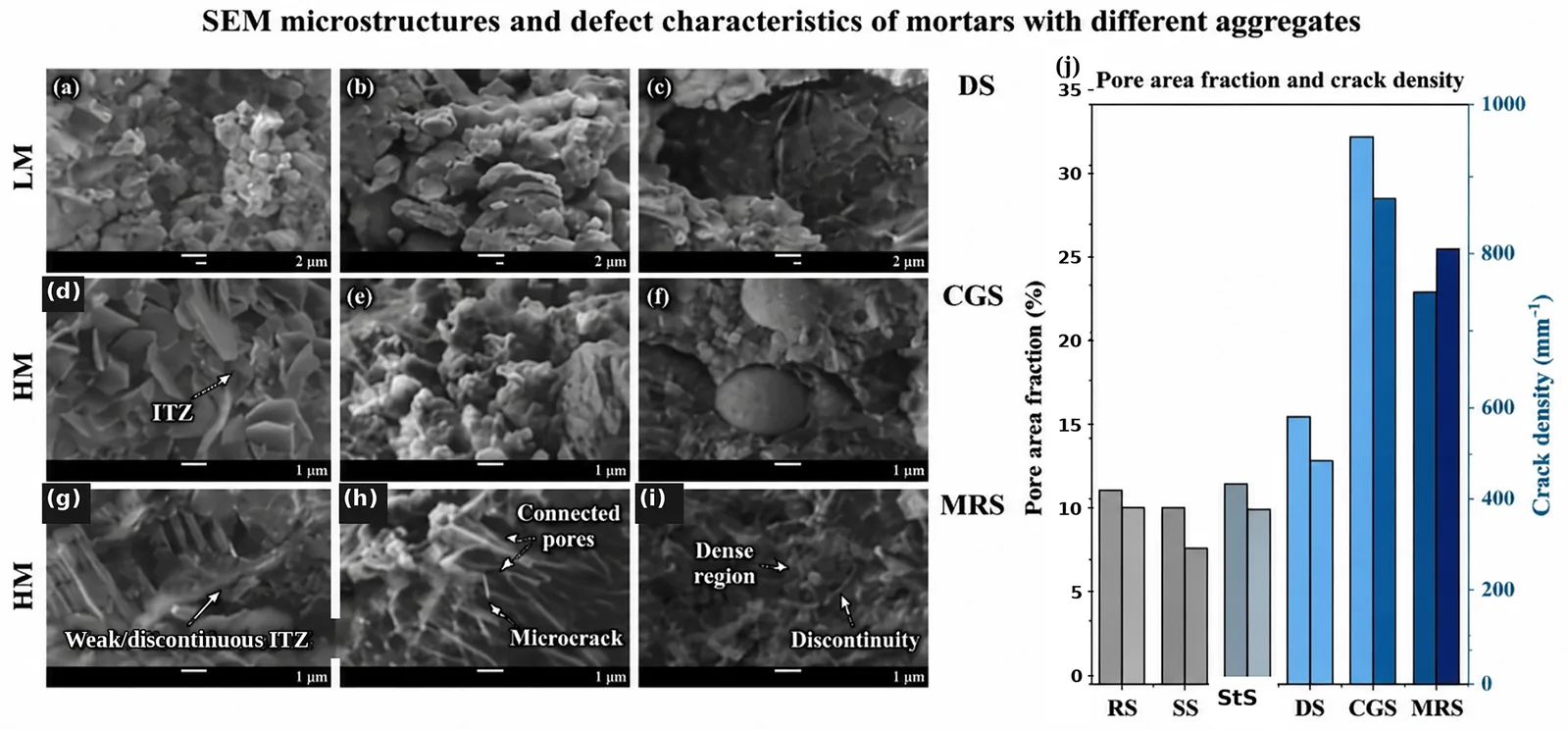
SEM microstructures and image-based defect descriptors of mortars containing different aggregates. Panels (**a**–**c**), (**d**–**f**), and (**g**–**i**) show representative fields from DS, CGS, and MRS mortars, respectively, at 28 d; LM and HM denote lower and higher magnification. Panel (**j**) reports the pore area fraction on the left axis (lighter bar in each pair) and the crack density on the right axis (darker bar) for representative fields from RS, SS, StS, DS, CGS, and MRS mortars. The chart describes the analyzed two-dimensional fields and must not be interpreted as describing bulk porosity or three-dimensional crack density.

The DS fields contained compact hydration-product regions together with isolated open pores. In CGS, the annotated ITZ was locally irregular, and porous clusters and rounded voids remained visible. MRS combined dense matrix regions with weak or discontinuous interfaces, connected pores, and crack-like features. The field-based descriptors in Figure 5j were higher for CGS and MRS than for the conventional reference mortars, and CGS showed the largest pore area fraction in the analyzed fields. These observations are consistent with interrupted local load paths, but they do not establish the origin or three-dimensional abundance of the defects.

### 4.2. Elemental Characteristics of the Raw MRS and CGS Aggregates

**Figure 6:**
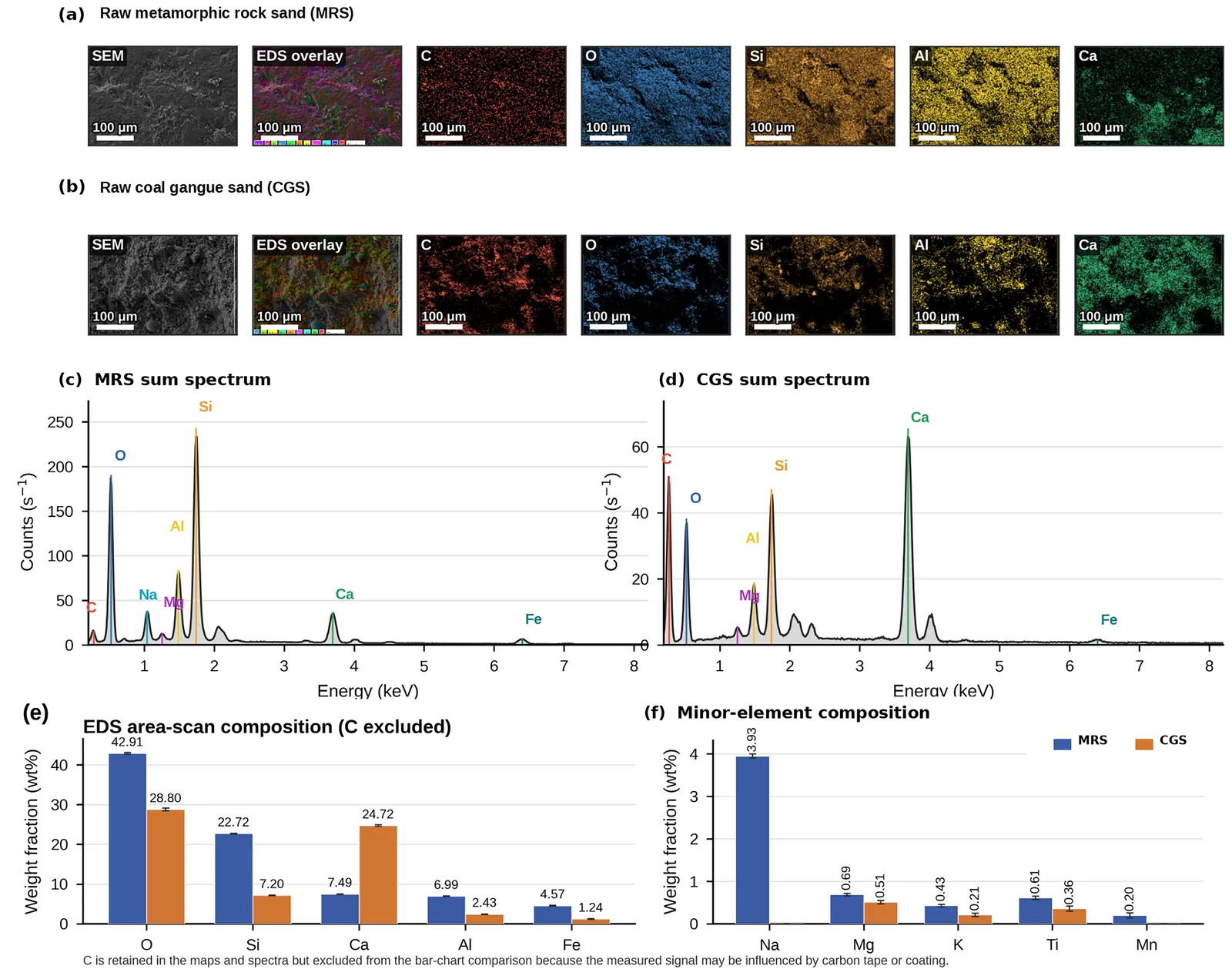
SEM-EDS characterization of raw MRS and CGS: (**a**) MRS electron image; EDS overlay; and C, O, Si, Al, and Ca maps. (**b**) Corresponding CGS images and maps. (**c**) MRS sum spectrum. (**d**) CGS sum spectrum. (**e**) Major-element area-scan composition, excluding C. (**f**) Minor-element composition. Error bars are the analytical 1σ uncertainties reported by the EDS software. Carbon was excluded from the bar charts because carbon tape or coating can influence the measured signal.

As shown in Figure 6, the analyzed MRS region contained 42.91 wt% O, 22.72 wt% Si, 7.49 wt% Ca, 6.99 wt% Al, and 4.57 wt% Fe, whereas the CGS region contained 28.80 wt% O, 7.20 wt% Si, 24.72 wt% Ca, 2.43 wt% Al, and 1.24 wt% Fe. The MRS field also contained 3.93 wt% Na, while Mg, K, Ti, and Mn were minor in both scans. The maps document local chemical heterogeneity and a Ca-rich domain in the selected CGS field. These elemental mass fractions cannot be compared numerically with the normalized bulk oxide mass fractions in Table 2 without an element-to-oxide conversion. They should not be generalized to the entire aggregate batch.

### 4.3. XRD Phase Evolution

**Figure 7:**
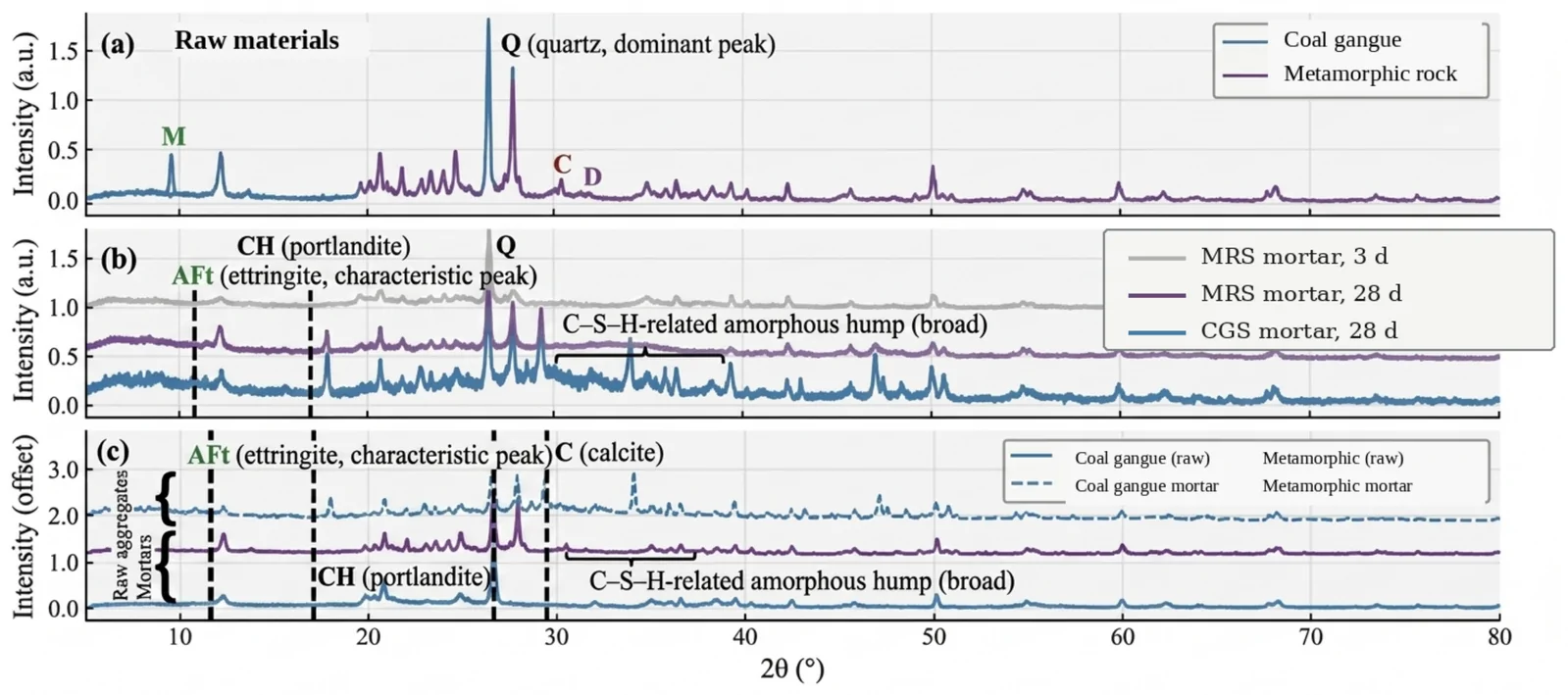
Qualitative XRD patterns of the selected aggregates and mortars: (**a**) raw CGS and raw MRS; (**b**) MRS mortar at 3 and 28 d together with CGS mortar at 28 d; and (**c**) vertically offset comparisons of the raw aggregates with their corresponding 28 d mortars. M, Q, D, C, CH, and AFt denote a mica-type phase, quartz, dolomite, calcite, portlandite, and ettringite, respectively. The bracketed diffuse region is associated with poorly crystalline C–S–H-containing binder and is not assigned as a discrete crystalline phase. Intensities are offset for clarity and are not interpreted as quantitative phase contents because no internal standard or Rietveld refinement was used.

The raw patterns were dominated by quartz but contained distinct minor reflections, including the annotated mica-type, calcite, and dolomite features (Figure 7a). The mortar patterns retained strong aggregate-derived quartz reflections and contained additional peaks assigned to portlandite and ettringite above a broad, diffuse background associated with poorly crystalline binder phases (Figure 7b). Changes in relative peak prominence between the 3 and 28 d MRS mortar patterns indicate age-dependent phase development during the interval, in which the measured strength passed through a 7 d maximum and declined by 28 d. Because the traces were offset and no quantitative phase analysis was performed, these differences do not provide reaction degrees or absolute phase contents.

Figure 7c compares each raw aggregate with its corresponding 28 d mortar and shows the persistence of aggregate-derived peaks alongside hydration-related features. Differences between the CGS and MRS mortars may also reflect aggregate dilution, preferred orientation, particle statistics, and the relative proportions of aggregate and paste collected during grinding. Conventional XRD does not resolve the spatial distribution or mechanical role of poorly crystalline C–S–H within the ITZ. The patterns therefore show that hydration products were present, but they do not identify the cause of the strength reversal and must be interpreted together with the microscopy.

Table 3 consolidates the evidentiary scope and principal limitations of each method.

## 5. Discussion

### 5.1. Reliability and Meaning of the Strength Reversal

The specimen-level loads and strengths in Supplementary Table S1 reproduce the approximately 8–65 MPa range reported in the manuscript. Equation (1) states the loaded area and the K=1.35 coefficient prescribed by Clause 9.0.4 of JGJ/T 70–2009. The coefficient was applied uniformly to all 54 specimens before grouping or statistical analysis. The supplier-provided cement strengths in Table 1 were obtained by a separate standard-mortar procedure and were not recalculated using Equation (1).

The decreases in CGS and MRS were large relative to the scatter among the three cubes tested at each age, and the exploratory Tukey test revealed significant differences. However, every aggregate was represented by only one mortar batch, from which the 3, 7, and 28 d specimens were allocated. The inferential statistics therefore characterize within-batch specimen variation and cannot demonstrate that the reversal would recur in an independently prepared batch. The absence of 14 and 21 d measurements is equally important: the data show that the 28 d value was lower than the 7 d value, but they cannot determine whether the decrease was gradual, abrupt, or associated with a particular handling or curing interval. The finding should be treated as a preliminary screening signal until it is reproduced in independent mixing batches with intermediate-age testing.

### 5.2. Moisture State, Porosity, Clay Content and Grading

The common nominal mixture design allows a direct screening comparison but does not isolate intrinsic aggregate effects. If the measured as-received moisture was not corrected, the water carried by the aggregate corresponded to approximately 0.0009–0.0264 kg per kilogram of cement across the six mixtures. The resulting total water-to-cement ratio before absorption would range from about 0.451 to 0.476 rather than remain exactly 0.45. At the same time, porous particles can remove water from the fresh paste. The net effect depends on absorption kinetics and cannot be inferred from total porosity alone.

CGS combined the highest measured porosity with the highest initial moisture content, conditions that may promote both water uptake and non-uniform internal moisture storage. MRS was much less porous than CGS but had the largest fineness modulus of all tested aggregates, along with a coarse, angular particle morphology that may reduce packing compatibility and create local stress concentrations. SS had the highest clay content but retained positive strength development after 7 d, although its absolute strength was low. These contrasts show that porosity and clay content act through different pathways and that neither property alone explains the negative SVI_7–28_ values.

Because saturated-surface-dry conditioning, absorption–time curves, flow-table spread, and air content were not measured, effective water availability and compaction remain credible alternative explanations. The present results should therefore be read as a screening outcome under a common nominal formulation, not as proof that aggregate mineralogy alone caused the strength reversal.

### 5.3. Limits of the Microstructural Evidence and Alternative Explanations

The available characterization does not have the age resolution required to trace a defect-formation process between 7 and 28 d. SEM was limited to selected fractured and dried 28 d fields; raw-aggregate EDS did not examine the hydrated ITZ; and XRD included MRS mortar at 3 and 28 d and CGS mortar at 28 d but no 7 d pattern and no CGS age series. These methods therefore document endpoint or contextual features but cannot show when a defect formed, whether it predated the 7 d maximum, or whether it caused the subsequent strength reduction.

Several alternative explanations remain unresolved, including aggregate moisture exchange, differences in workability and entrapped air, packing and compaction, shrinkage-related cracking, phase-related dimensional change, and ordinary mixing or casting variability. The crack-like features in the SEM fields may also have been created or enlarged during fracture and drying. None of these possibilities was measured with sufficient temporal or replicate resolution to assign a material-specific mechanism to CGS or MRS. The microstructural observations are therefore hypothesis-generating only and should not be used as proof of a competition process between hydration and defects.

### 5.4. Practical Implications and Future Work

A single early-age strength result is insufficient for screening an unfamiliar fine aggregate. The 7 d strength and SVI_7–28_ should be considered together, with absolute strength and durability requirements applied separately. A negative retention value should trigger additional testing rather than automatic acceptance or rejection.

A more rigorous qualification program should condition aggregates to defined moisture states, measure short-term absorption, adjust batch water, and verify equal or intentionally controlled flow and air content. Mechanical testing should include 14 and 21 d ages, more specimens, and independently prepared batches. Quantitative microstructure should combine polished backscattered-electron imaging, mercury intrusion porosimetry or gas adsorption, X-ray computed tomography, thermogravimetry, quantitative XRD, and nanoindentation. Shrinkage, expansion, internal relative humidity, and pore-solution chemistry would help separate moisture-driven, phase-related, and interfacial causes [14,29,30,31,32].

## 6. Conclusions

In this preliminary single-batch screening study, mortars containing RS, StS, DS, and SS gained strength between 7 and 28 d, whereas the tested CGS and MRS batches showed within-batch strength reversal. CGS decreased from 15.56±0.31 to 11.68±0.15 MPa and MRS from 21.63±0.48 to 15.75±0.20 MPa, giving SVI_7–28_ values of −24.95% and −27.18%, respectively. These specimen-level triplicates do not establish batch-to-batch reproducibility.

Negative retention occurred in aggregates with very different measured porosities. CGS had the highest bulk porosity, while MRS was comparatively dense. SS had the highest clay content but remained in the positive-retention group. The observed behavior therefore cannot be attributed to porosity or clay content alone.

The selected 28 d SEM fields contained pores, interfacial discontinuities, and crack-like features, while raw-aggregate EDS and qualitative XRD provided contextual chemical and phase information. However, SEM was not age-resolved, EDS did not characterize the hydrated ITZ, and XRD lacked a 7 d pattern and a CGS age series. The measurements therefore cannot reconstruct defect formation between 7 and 28 d or establish a causal mechanism for the strength reversal.

The principal practical implication is that an unexpected negative strength-retention result should trigger confirmatory testing rather than immediate mechanistic interpretation. Defined moisture conditioning, control of effective water content, flow and air-content measurements, intermediate ages, and independently prepared replicate batches are required before the screening signal can be generalized or translated into mixture-design recommendations.

## Figures and Tables

**Table 1:** Chemical composition and physical properties of cement.

Chemical Composition (Mass %)		Physical Properties	
Component	Value	Property	Value
SiO_2_	21.86	Specific surface area (m^2^/kg)	342.0
Al_2_O_3_	5.42	Standard consistency (%)	27.8
Fe_2_O_3_	3.71	Initial setting time (min)	146
CaO	61.35	Final setting time (min)	275
MgO	3.68	3 d compressive strength (MPa)	24.6
SO_3_	2.64	28 d compressive strength (MPa)	46.8

The reported cement strengths are supplier-provided standard-mortar results and were not recalculated using Equation (1); the coefficient K=1.35 was not applied to these supplier values. They are not directly comparable to the 70.7 mm cube strengths reported in this study because the specimen geometry and calculation procedure differ.

**Table 2:** Normalized chemical compositions and physical properties of the fine aggregates.

Item	SS	RS	StS	DS	CGS	MRS
Chemical composition (mass %)						
SiO_2_	60.07	91.10	98.92	72.83	56.66	62.79
Al_2_O_3_	17.95	3.38	0.35	7.42	22.67	16.59
Fe_2_O_3_	5.53	0.54	0.10	2.29	5.28	4.56
FeO	1.24	0.51	0.06	0.83	1.18	1.40
CaO	4.62	1.03	0.08	8.60	3.03	5.09
MgO	2.79	0.19	0.04	2.04	2.76	3.05
K_2_O	2.63	1.94	0.12	1.95	2.54	2.04
Na_2_O	1.36	0.25	0.05	2.41	1.09	1.22
Loss on ignition	3.81	1.06	0.28	1.63	4.79	3.26
Total	100.00	100.00	100.00	100.00	100.00	100.00
Physical properties						
Apparent density (kg/m^3^)	2580	2610	2670	2630	2520	2650
Bulk density (kg/m^3^)	1289.90	1390.16	1714.68	1551.62	1111.42	1631.36
Porosity (%)	49.9	46.7	35.8	41.0	55.9	38.4
Clay content (%)	8.6	2.1	0.2	1.8	3.8	4.5
Moisture content (%)	0.53	0.29	0.08	0.03	0.92	0.38
Fineness modulus	2.15	2.60	2.50	1.20	2.41	2.75
Chloride ion content (%)	0.03	0	0	0	0.02	0.01
Sulfate content (%)	0.20	0.14	0.03	0.10	0.18	0.12

Note: The oxide results were normalized to 100 mass% on a common total-mass basis that includes loss on ignition. The displayed values are rounded. These bulk results characterize the raw aggregates and should not be interpreted as local ITZ compositions.

**Table 3:** Scope and limitations of the characterization methods.

Method	Samples	Supported Observation	Interpretation Limit
Strength	Six mortars; one batch per aggregate; n=3 cubes per age at 3, 7, and 28 d	Within-batch reductions in CGS and MRS from 7 to 28 d	Specimen-level triplicates do not establish batch-to-batch reproducibility and do not resolve the path between 7 and 28 d
SEM	Selected 28 d DS, CGS, and MRS fields	Local pores, dense regions, discontinuous interfaces, and crack-like features	Fracture and drying can alter morphology; fields selected at 28 d do not represent bulk three-dimensional structure or track defect formation from 7 to 28 d
Image analysis	Representative fields from six mortars	Two-dimensional pore-area and crack-length descriptors	No replicate-field statistics or age sequence; not equivalent to bulk porosity or material-level crack density
EDS	Raw MRS and CGS	Local elemental distributions and surface composition	Elemental and bulk-oxide bases are not directly comparable; no hydrated-ITZ chemistry, phase identification, or bond measurement
XRD	Raw CGS/MRS; MRS mortar at 3 and 28 d; CGS mortar at 28 d	Crystalline assemblages, hydration-related reflections, and qualitative age-related changes	No 7 d pattern or CGS age series; no quantitative phase content, reaction degree, amorphous content, or ITZ-specific distribution

## Data Availability

The original contributions presented in this study are included in the article/Supplementary Material. Further inquiries can be directed to the corresponding author.

## Supplementary Materials

The following supporting information can be downloaded at: https://www.mdpi.com/article/10.3390/ma19163437/s1, Table S1: Individual failure loads, specimen identifiers, batch identifiers, loaded area, conversion coefficient, and calculated compressive strengths for all 54 cubes used in the manuscript.
